# CCNB1 is a novel prognostic biomarker and promotes proliferation, migration and invasion in Wilms tumor

**DOI:** 10.1186/s12920-023-01627-3

**Published:** 2023-08-17

**Authors:** Bin Xiang, Mei-Lin Chen, Zhi-Qiang Gao, Tao Mi, Qin-Lin Shi, Jun-Jun Dong, Xiao-Mao Tian, Feng Liu, Guang-Hui Wei

**Affiliations:** 1 Ministry of Education Key Laboratory of Child Development and Disorders, Chongqing Key Laboratory of Pediatrics, National Clinical Research Center for Child Health and Disorders, International Science and Technology Cooperation Base of Child Development and Critical Disorders, Children’s Hospital of Chongqing Medical University, Chongqing, P.R. China; 2 Department of Urology, Chongqing Key Laboratory of Children Urogenital Development and Tissue Engineering, Children’s Hospital of Chongqing Medical University, Chongqing, P.R. China

**Keywords:** RNA sequencing, Wilms tumor, CCNB1, Prognoses, Nomogram, Immune infiltration

## Abstract

**Background:**

Wilms tumour (WT) is a mixed type of embryonal tumour that usually occurs in early childhood. However, our knowledge of the pathogenesis or progression mechanism of WT is inadequate, and there is a scarcity of beneficial therapeutic strategies.

**Methods:**

High-throughput RNA sequencing was employed in this study to identify differentially expressed genes (DEGs) in clinical tumor samples and matching normal tissues. The STRING database was utilized to build a protein-protein interaction (PPI) network, and the Cytohubba method was used to identify the top 10 highly related HUB genes. Then, the key genes were further screened by univariate COX survival analysis. Subsequently, the XCELL algorithm was used to evaluate the tumour immune infiltration. RT-PCR, WB, and IF were used to verify the expression level of key genes in clinical tissues and tumour cell lines. Finally, the function of the key gene was further verified by loss-of-function experiments.

**Results:**

We initially screened 1612 DEGs, of which 1030 were up-regulated and 582 were down-regulated. The GO and KEGG enrichment analysis suggested these genes were associated with ‘cell cycle’, ‘DNA replication’. Subsequently, we identified 10 key HUB genes, among them CCNB1 was strongly related to WT patients’ overall survival. Multiple survival analyses showed that CCNB1 was an independent indicator of WT prognosis. Thus, we constructed a nomogram of CCNB1 combined with other clinical indicators. Single gene GSEA and immune infiltration analysis revealed that CCNB1 was associated with the degree of infiltration or activation status of multiple immune cells. TIDE analysis indicated that this gene was correlated with multiple key immune checkpoint molecules and TIDE scores. Finally, we validated the differential expression level of CCNB1 in an external gene set, the pan-cancer, clinical samples, and cell lines. CCNB1 silencing significantly inhibited the proliferation, migration, and invasive capabilities of WIT-49 cells, also, promoted apoptosis, and in turn induced G2 phase cell cycle arrest in loss-of-function assays.

**Conclusion:**

Our study suggests that CCNB1 is closely related to WT progression and prognosis, and serves as a potential target.

**Supplementary Information:**

The online version contains supplementary material available at 10.1186/s12920-023-01627-3.

## Introduction

Wilms tumour (WT) is a mixed type of embryonal tumour that accounts for more than 7% of childhood malignancies and 90% of pediatric renal tumours [1]. WT usually occurs in early childhood, and most cases are sporadic, with only 1-2% of familial cases [2]. It is well known that the tumour grows rapidly with the main clinical manifestation of a huge abdominal mass. The recurrence rate of WT is nearly 15%, and the long-term survival rate is only 50% [3]. As medical treatments have advanced, radical resection, radiotherapy, and chemotherapy have improved the 5-year survival rate to over 90% [4]. In addition, the side effects of high-intensity radiotherapy and chemotherapy seriously affect the quality of life of WT patients [5, 6]. However, our understanding of the pathogenesis and progression mechanisms of Wilms tumor is still insufficient, and there is a lack of effective therapeutic targets [7–10]. Hence, it is critical to investigate the molecular processes behind WT development and to establish efficient biomarkers for better WT therapy and overall prognosis.

**Fig. 1:**
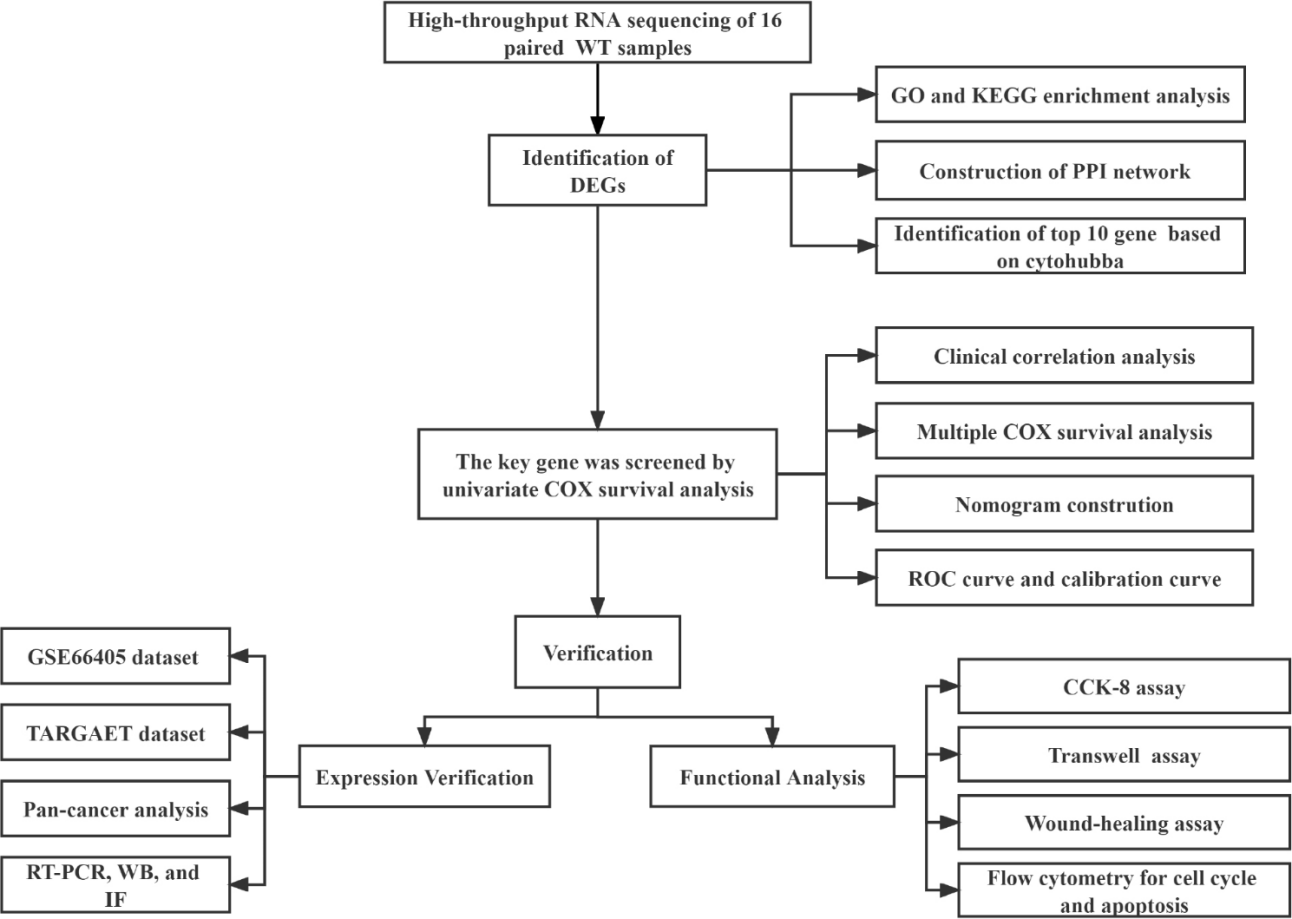
Flow chart of analysis for the present study

Since its first appearance in 2005 [11], high-throughput sequencing has become widely available with the popularization of sequencing technologies, and RNA-seq has become one of the most common applications of sequencing technology [12]. Differential gene expression analysis is one of the primary applications of RNA-seq, which can reveal the functional and potential molecular mechanisms of differentially expressed genes identified [13]. Importantly, differential gene expression analysis is useful for identifying potential biomarkers for cancer [14]. Bioinformatics has been widely applied to various cancer studies and has been proven effective and reliable for diagnosing cancer and targeting new tumor markers [15]. Many cancers, including Wilms’ tumor, occur due to genetic mutations. Using bioinformatics methods, we can identify and validate key genes related to cancer and provide new targets for diagnosis and treatment [16]. In this study, we used high-throughput sequencing combined with bioinformatics methods to screen key genes associated with the prognosis of Wilms’ tumor and verified their specific expression and impact on the malignant biological phenotype of Wilms’ tumor through a series of basic experiments. Figure 1 displays our main steps.

## Materials and methods

### Clinical specimens

All programs were approved by the Ethics Committee of the affiliated Children’s Hospital of Chongqing Medical University and all patients diagnosed with WT had not received radiotherapy or chemotherapy before surgery. We collected WT tissue and normal kidney tissue from 37 patients who underwent surgery from January 2019 to September 2022 (Table 1). Each sample was immediately frozen in liquid nitrogen and preserved. All samples were identified as WT by pathology tissue department specialists, and all WT samples were classified by pathologists following the Children Oncology Group (COG) staging system [17]. We randomly selected 8 pairs of tumor samples and paired normal tissue samples for mRNA sequencing to obtain the expression data of mRNA in WT. The rest of the samples were used for gene expression validation.

**Table 1:** The clinicopathological features of WT patients. WT, Wilms tumor; TNM, tumor-node-metastasis; FH, favorable histology; UH, unfavorable histology

Characteristics	Total (N = 37)
Gender	
Female	19 (51.35%)
Male	18 (48.65%)
Age (years)	
> 3year	11 (29.73%)
≤ 3year	26 (70.27%)
TNM	
I-II	13 (35.14%)
III-V	24 (64.86%)
Pathologic types	
UH	16 (43.24%)
FH	21 (56.76%)

### RNA sequencing (RNA-seq) of WT tissue

The Illumina NovaSeq 6000 was used for library construction and sequencing at Shanghai Sinomics Corporation (Illumina, USA). Detailed procedures can be referred to the previously published paper [18].

### Identification of DEGs

To measure mRNA levels, we utilized fragments per kilobase million (FPKM) to reflect the expression of various genes, which could be determined as follow:

$$\text{F}\text{P}\text{K}\text{M}=\frac{\text{t}\text{o}\text{t}\text{a}\text{l} \text{e}\text{x}\text{o}\text{n} \text{F}\text{r}\text{a}\text{g}\text{m}\text{e}\text{n}\text{t}\text{s}}{\text{m}\text{a}\text{p}\text{p}\text{e}\text{d} \text{r}\text{e}\text{a}\text{d}\text{s} \left(\text{M}\text{i}\text{l}\text{l}\text{i}\text{o}\text{n}\text{s}\right)\times \text{e}\text{x}\text{o}\text{n} \text{l}\text{e}\text{n}\text{g}\text{t}\text{h} \left(\text{K}\text{B}\right)}$$

The fragments within each gene interval were first counted using Stringtie software [19]. Finally, FPKM values were then calculated for each gene, and the software edgeR [20] was used to analyze the differences in gene expression between groups. The final P-value was calculated for differential expression between tumour and normal tissues. Meanwhile, the differential expression fold change (FC) was calculated based on the FPKM values. Ultimately, DEGs were evaluated using both |logFC| > 1 and Q-value 0.05 as filtering principles.

### Enrichment Analysis and hub genes identification

To investigate the underlying mechanics of DEGs in WT, we used the Gene Ontology (GO) and the Kyoto Encyclopedia of Genes and Genome (KEGG) [21] to explore potential biological processes and pathways using the R package ‘clusterProfiler’ [22]. In addition, DEGs were uploaded to the STRING database [23] to construct a protein-protein interaction (PPI) network. Cytoscape [24] based on the Cytohubba algorithm [25] was adopted to identify the top ten highly correlated HUB genes.

### **Gene Set Enrichment Analysis** (**GSEA)**

GSEA is a functional annotation tool for understanding the underlying biological pathways of different biological phenotypes or states [26]. In this study, we performed GSEA analysis using gene sets based on GO, KEGG, and ImmuneSigDB. We grouped the samples based on the median expression of CCNB1 and identified the biological processes and pathways that were significantly associated with high and low expression groups.

### Survival analysis

The TARGET database was used to acquire gene transcription and patient information on WT individuals. To compare survival differences across groups, log-rank was used to assess the Kaplan-Meier (KM) survival analysis, and time-dependent receiver operating characteristic (ROC) curve analysis was done to predict accuracy. The survival outcome metric used was overall survival (OS). We conducted a multivariate Cox survival analysis of CCNB1 and other clinical indicators, resulting in three independent prognostic factors: stage, gender, and CCNB1. We then constructed a risk score (Riskscore) based on the impact of each indicator on prognosis in the COX regression model, and divided the subjects into high-risk and low-risk groups based on the median of the risk score for further survival analysis. Subsequently, the independent prognostic value of CCNB1 was constructed and evaluated in conjunction with clinical indicators.

### Assessment of Immune Infiltration

The Stromalscore and Immunescore, which reflect the extent of TME-related cell infiltration in WT tumor tissue, were calculated using the “IOBR” R package, employing the ESTIMATE algorithm[27, 28]. The ESTIMATE algorithm was developed based on single-sample gene set enrichment analysis (ssGSEA). The TME consists of various types of immune cells. To assess the heterogeneous cellular landscape of the TME, cell type enrichment scores were evaluated. In the TARGET and GSE31403 datasets, the XCELL method [29] was used to calculate the degree of immune cell infiltration and immune score. In addition, in the TARGET cohort, we employed the Tumour Immune Dysfunction and Exclusion (TIDE) score to measure treatment responsiveness to immune checkpoint inhibitors [30].

### Expression Verification

We obtained and downloaded the WT expression dataset (GSE66405) from the GEO platform for expression validation of CCNB1. Subsequently, a uniformly normalized pan-cancer dataset was downloaded from the Sangerbox platform (http://sangerbox.com/home.html), from which the expression data of the CCNB1 (ENSG00000134057) gene in each sample were extracted. Meanwhile, expression differences in clinical tissues and cell lines were calculated using an unpaired t-test.

#### RT-PCR

Tissue RNA was extracted from previously collected WT tissue and normal kidney tissue in 37 patients. Cellular RNA was extracted from WT cells (WIT-49) and normal renal epithelial cells (293T). RT-PCR methodology was performed according to previous literature [18]. The expression of CCNB1 and GAPDH was based on the formula 2^^-ΔΔCt^. Table 2 lists the primers that were utilized.

**Table 2:** Primers used for quantitative real time PCR.

RNA	Forward primer Sequence (5`−3`)	Reverse primer Sequence (5`−3`)
CCNB1	TTTCTGCTGGGTGTAGGTCC	GCCATGTTGATCTTCGCCTT
GAPDH	CCTTCCTGGGCATGGAGTC	TGATCTTCATTGTGCTGGGTG

### Cell transfection

Table 3 shows the CCNB1 targeting siRNA and negative control (NC) siRNA sequences (TSINGKE, Beijing, China). The WIT-49 cells were plated onto 6-well or 12-well plates and divided into four groups: experimental group for CCNB1siRNA-1, CCNB1siRNA-2, CCNB1siRNA-3, and negative control group with NC siRNA. Lipofectamine™ RNAiMAX (Invitrogen, USA) was used for transient transfection according to the manufacturer’s instructions.

**Table 3:** CCNB1 siRNA sequences

siRNA	Forward primer Sequence (5`−3`)	Reverse primer Sequence (5`−3`)
CCNB1 siRNA−1	GCUGAAUUCUGCACUAGUUTT	AACUAGUGCAGAAUUCAGCTT
CCNB1 siRNA−2	GGUAAAUCAAGGACUUACATT	UGUAAGUCCUUGAUUUACCTT
CCNB1 siRNA−3	CUGACAACACUUAUACUAATT	UUAGUAUAAGUGUUGUCAGTT

* The siRNA NC was provided in the reagent

### Western blotting and immunofluorescence

The previous article [31] described experimental methods for western blot (Prior to antibody hybridization, we cut the membrane at corresponding band positions.) and immunofluorescence. The following antibodies were used in the experiments: primary antibody (Anti-CCNB1 Rabbit pAb, GB112098; Anti-GAPDH Rabbit pAb, GB11002, Servicebio, China), secondary antibody (HRP − conjugated secondary antibody, G1213-100UL, Servicebio, China).

### Cell proliferation, Migration, and Invasion Assay

The CCK-8 counting method (MCE, HY-K0301) was used to determine cell viability. Approximately 1 × 10^4^ WIT-49 cells were inoculated into 96-well plates and transfected with CCNB1siRNA-1 and NCsiRNA. Transwell invasion assay to determine cell invasion ability. A scratch wound healing motility assay was performed to determine cell migration using WIT-49 cells. A methodology was referenced from the previous literature of our team [32].

### Flow Cytometry

Flow cytometry was used to determine the distribution of cell cycle phases and the amount of apoptosis using the BD detection kit. FlowJo software was used to handle all data.

### Statistical analysis

The experimental data were analyzed and graphed using the statistical program GraphPad Prism 8. Sangerbox was used for all bioinformatics analyses. Every experiment was carried out three times. A *p*-value less than 0.05 was considered statistically significant.

## Results

### Identification of DEGs in WT tissue and functional Enrichment Analysis

**Fig. 2:**
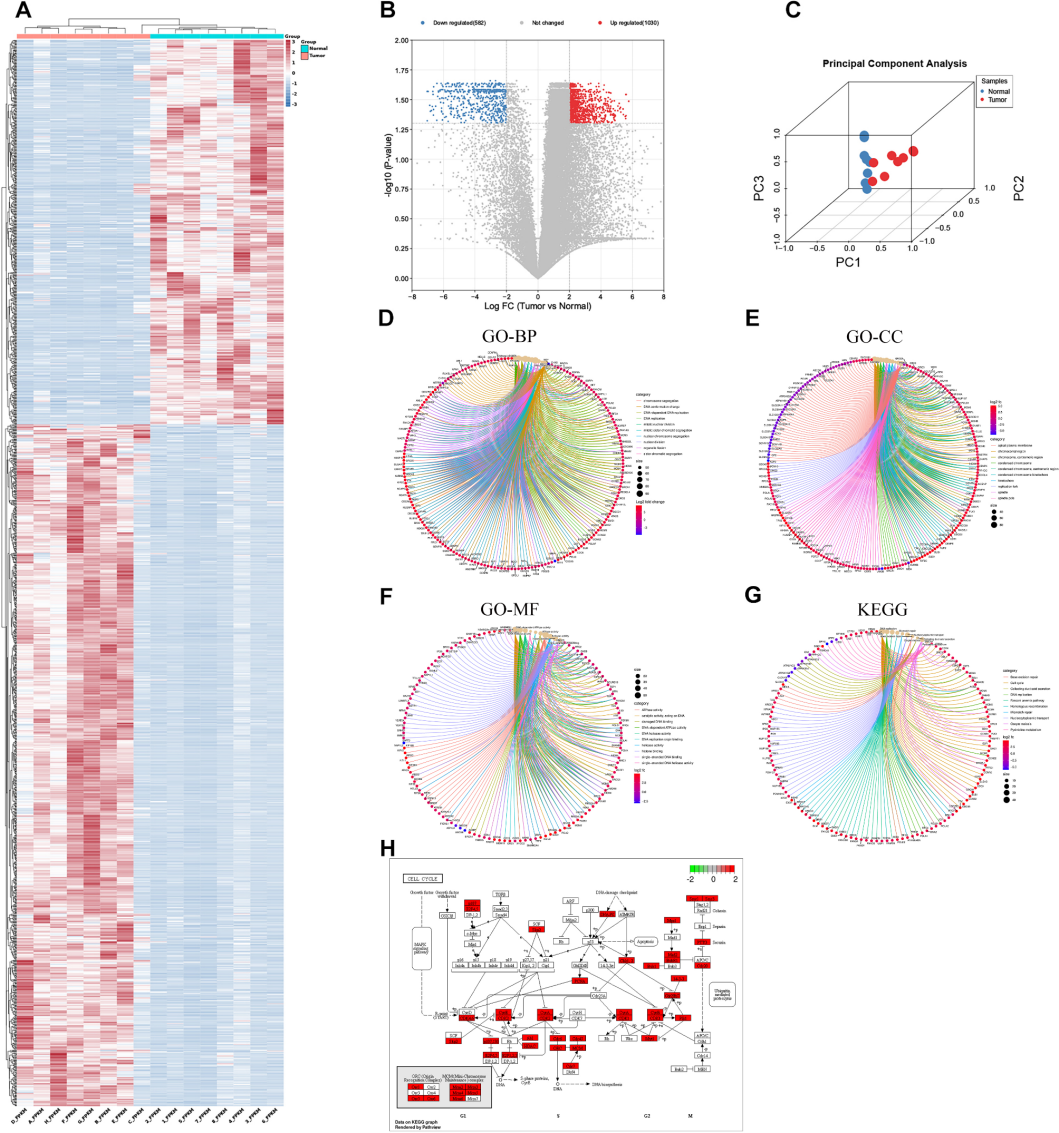
**Identification of DEGs in WT Tissue and Functional Enrichment Analysis. (A)** Heatmaps and cluster analysis of DEGs. Each row represents a gene, and each column represents a sample. Red indicates high expression, while blue indicates low expression. Letters represent tumor samples, and numbers represent normal samples (paired as follows: A-1, B-2, C-3, D-4, E-5, F-6, G-7, H-8). **(B)** Volcano plot of DEGs. Red represents upregulated genes (1030), blue represents downregulated genes (582). The horizontal dashed line represents an adjusted P-value of 0.05, and the vertical dashed line represents a logFC of 2. **(C)** PCA analysis shows a remarkable difference in transcriptomes between the tumors (red) and normal tissues (blue). **(D-H)** GO and KEGG enrichment analysis showed that DEGs were mainly enriched in biological pathways or processes such as ‘DNA replication’, ‘cell cycle’

We conducted mRNA sequencing analysis on 8 pairs of tumor samples and matched normal tissue samples, identifying distinct mRNA expression profiles in WT. According to the method’s guideline, a total of 1612 differentially expressed genes were revealed, with 1030 being up-regulated and 582 being down-regulated (Fig. 2B). Cluster heat map and PCA analysis showed that differential genes distinguished the tumour from normal tissue (Fig. 2A, C). Following GO and KEGG enrichment analysis, the DEGs were shown to be significantly enriched in biochemical processes such as ‘DNA replication’, ‘Cell cycle’. (Fig. 2D-H).

### PPI Network Construction and hub genes identification

**Fig. 3:**
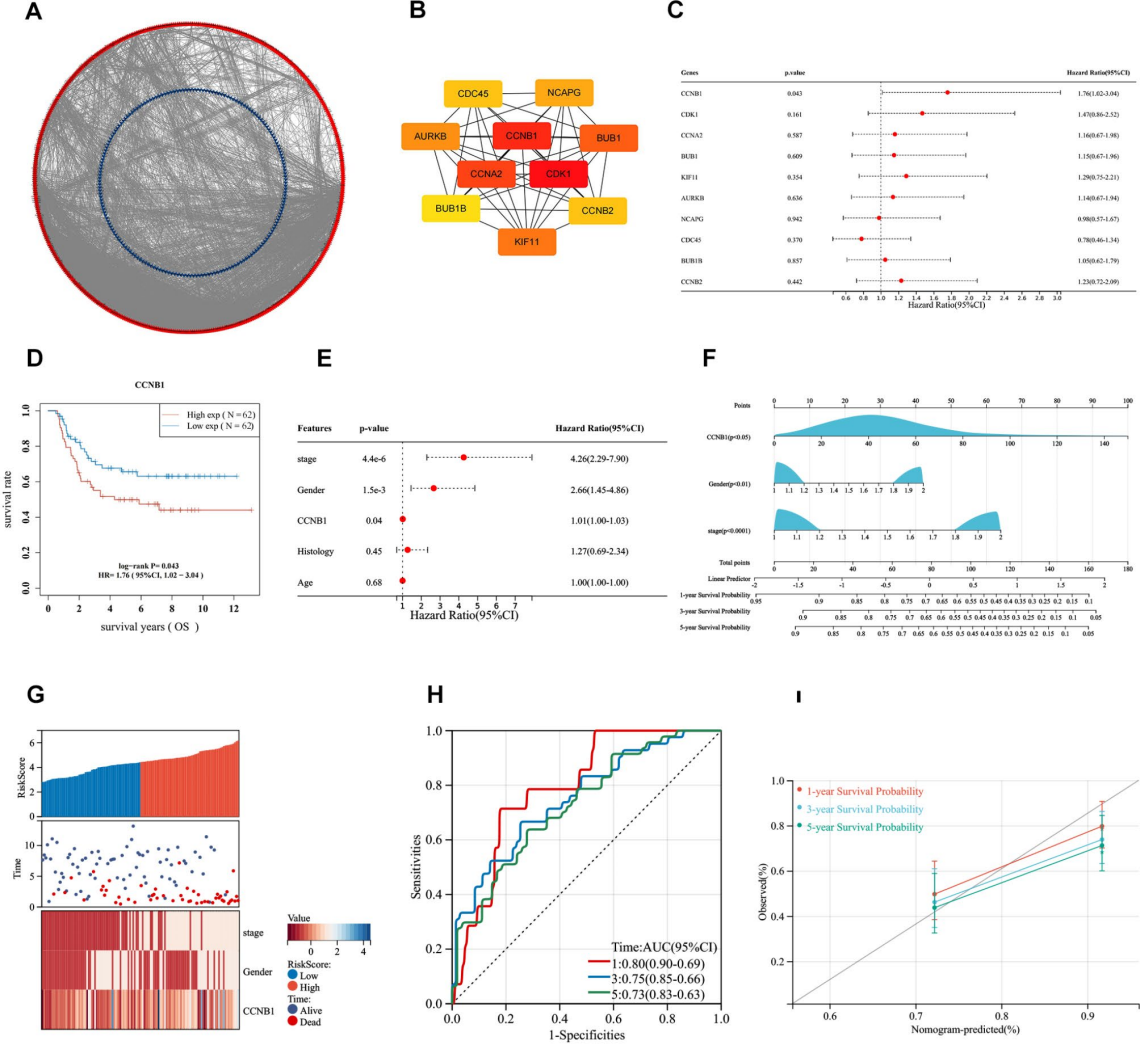
**Identification of prognosis-related HUB genes and construction of a nomogram. (A)** A PPI network of 1612 DEGs based on the STRING database. Red represents upregulated genes, blue represents downregulated genes. **(B)** Identification of top ten Hub genes using cytoHubba algorithm. **(C)** Univariate Cox analysis of the 10 Hub genes showed that CCNB1 was significantly correlated with OS in WT patients. **(D)** KM survival curves show that high expression of CCNB1 was associated with poorer prognosis in WT. **(E)** Multiple COX regression analyses revealed that CCNB1 was a critical risk biomarker independent of clinical indicators. **(F)** Combining CCNB1 expression and clinical indicators to develop a nomogram that could predict patient survival (1-, 3-, and 5-year survival rates). **(G)** Combined CCNB1 expression and clinical indicators to assess patient risk scores. **(H and I)** ROC curve and correction curve showed that the prediction model had good prediction performance (1-, 3-, and 5-year survival rates)

We constructed a PPI network of 1612 DEGs based on the STRING database. The PPI network includes 693 nodes and 6656 edges (Fig. 3A). We used cytoHubba to obtain the top 10 most connected Hub genes in the PPI network, including CCNB1, CDK1, CCNB2, CCNA2, BUB1, KIF11, AURKB, NCAPG, CDC45, and BUB1B (Fig. 3B).

### Survival and clinical relevance analysis

Univariate Cox analysis of 10 Hub genes showed that CCNB1 was significantly associated with OS of WT patients (Fig. 3C, D). Clinical correlation showed that the expression of CCNB1 was significantly associated with the pathological type and tumour progression (Table 4), suggesting that the gene may be involved in the malignant progression of WT. Multiple survival analyses showed that CCNB1 was an independent predictor of WT patients. Therefore, we constructed a nomogram (the C-index of the nomogram was 0.72) by combining other clinical indicators for predicting the near and long-term survival of patients (Fig. 3E, F). Subsequently, we calculated risk scores for each patient in a composite model with multiple indicators (Fig. 3G). The ROC curves and calibration curves showed good performance of the prediction model. The AUC values predicting 1-, 3-, and 5-year survivals were increased to 0.80, 0.75, and 0.73, respectively (Fig. 3H, I).

**Table 4:** Correlation between the expression of CCNB1 and the clinicopathological features of WT patients

	CCNB1 expression (%)			
Characteristics	Low (N = 62)	High (N = 62)	Total (N = 124)	*P*
Gender				0.20
Female	39 (31.45%)	31 (25.00%)	70 (56.45%)	
Male	23 (18.55%)	31 (25.00%)	54 (43.55%)	
Race				0.44
Black or African American	7 (5.65%)	12 (9.68%)	19 (15.32%)	
Not Reported	3 (2.42%)	5 (4.03%)	8 (6.45%)	
Other	2 (1.61%)	3 (2.42%)	5 (4.03%)	
White	50 (40.32%)	42 (33.87%)	92 (74.19%)	
Age (years)				0.68
> 3year	44 (35.48%)	47 (37.90%)	91 (73.39%)	
≤ 3year	18 (14.52%)	15 (12.10%)	33 (26.61%)	
State				0.03
None	8 (6.45%)	19 (15.32%)	27 (21.77%)	
Progression	54 (43.55%)	43 (34.68%)	97 (78.23%)	
TNM				0.37
I-II	36 (29.03%)	30 (24.19%)	66 (53.23%)	
III-V	26 (20.97%)	32 (25.81%)	58 (46.77%)	
Pathologic types				< 0.001
UH	10 (8.06%)	32 (25.81%)	42 (33.87%)	
FH	52 (41.94%)	30 (24.19%)	82 (66.13%)	

WT, Wilms tumor; TNM, tumor-node-metastasis; FH, favorable histology; UH, unfavorable histology

### Analysis of the CCNB1-Associated Immune Infiltration

**Fig. 4:**
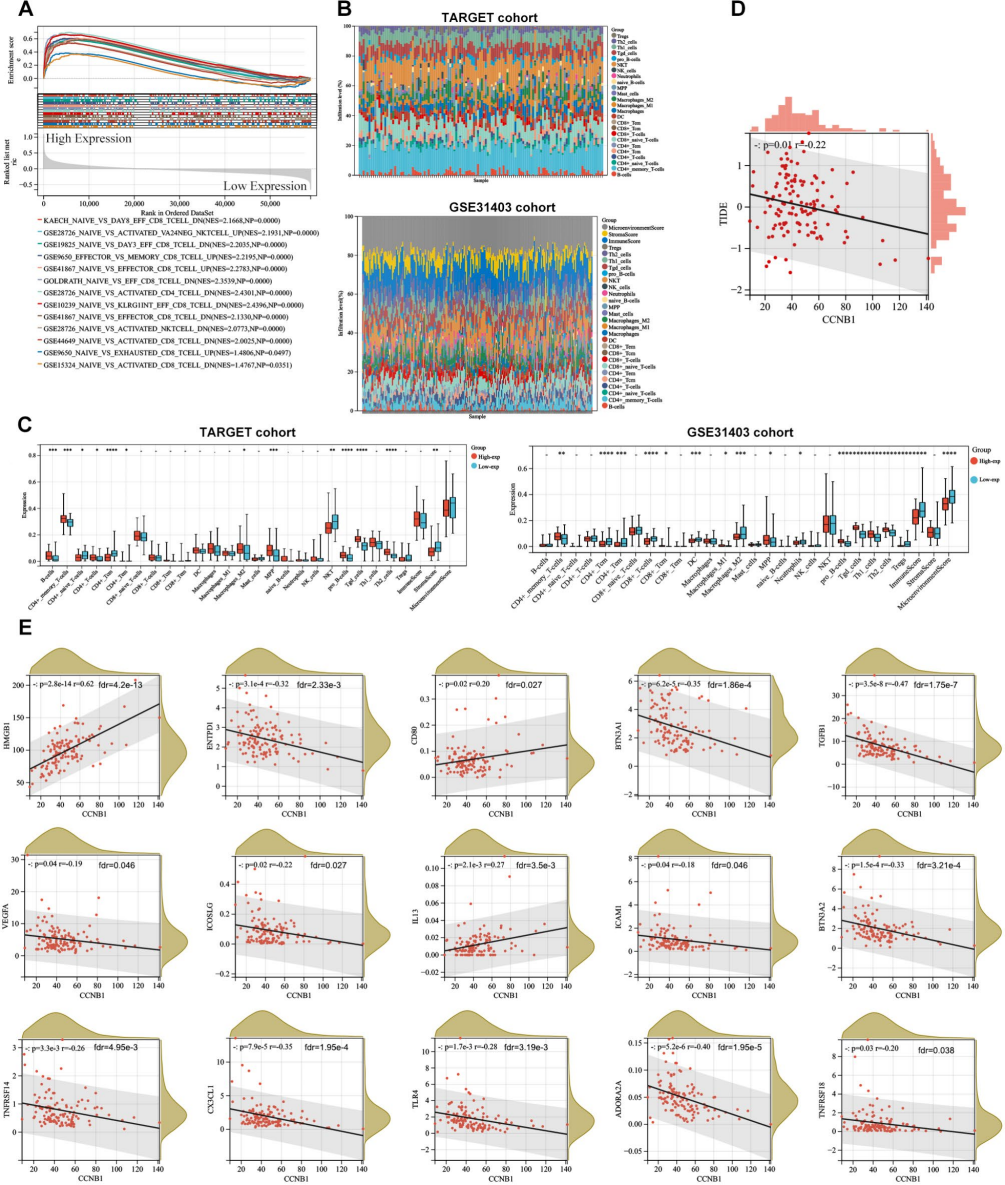
**Associations between CCNB1 expression and immune infiltration in TME. (A)** single-gene GSEA enrichment analysis showed that CCNB1 was involved in regulating the activation and function of immune cells. **(B)** Single-sample gene set enrichment analysis to generate heat maps to display immune scores and relative abundance of immune cell subsets in TARGET and GSE31403 datasets. **(C)** XCELL algorithm was used to calculate the ImmuneScore, StromaScore, MicroenvironmentScore, and immune cell infiltration levels between CCNB1 high expression subsets and low expression subsets in TARGET and GSE31403 cohorts. **(D and E)** CCNBI was correlated with the TIDE score and several immune checkpoint molecules. * *p* < 0.05, ** *p* < 0.01, *** *p* < 0.001, **** *p* < 0.0001

Single gene GSEA enrichment analysis revealed that this gene may be involved in regulating the activation and functional levels of multiple immune cells such as CD8^+^ T cells, CD4^+^ T cells, and NKT cells (Fig. 4A). Therefore, heat maps were generated by Single-sample gene set enrichment analysis (ssGSEA) to show the immune scores and relative abundance of immune cell subpopulations in the TARGET and GSE31403 datasets (Fig. 4B). The XCELL algorithm was used to calculate the ImmuneScore, StromaScore, MicroenvironmentScore, and immune infiltration level between high and low-expression subgroups of CCNB1 in the TARGET and GSE31403 cohorts (Fig. 4C). Compared to the low expression group in the TARGET cohort, the high CCNB1 expression group had a lower StromaScore. Furthermore, in the TARGET cohort, there was a significant increase in CD4 + memory T cells, CD4 + T cells, M2 macrophages, pro-B cells, γδ T cells, and Th2 cells in the CCNB1 high expression group. However, CD4 + naive T cells, CD4 + central memory (Tcm), and natural killer T (NKT) cells showed a significant increase in the CCNB1 low expression group. To explain the survival differences found in patients with good prognosis from an immune perspective, we further analyzed the differences in immune cell infiltration between CCNB1 high and low expression groups of WT patients in the GSE31403 cohort. In the GSE31403 cohort, the high expression group had lower ImmuneScore and MicroenvironmentScore compared to the low expression group. At the same time, the CCNB1 high expression group showed a significant increase in CD4 + memory T cells, CD8 + Tcm, pro-B cells, γδ T cells, and Th2 cells. The main lymphocyte subgroups involved in anti-tumor immunity, including CD4 + Tcm, CD4 + Tem, and CD8 + T cells, showed a significant increase in the CCNB1 low expression group. Taken together, these results suggest that the favorable prognosis associated with low CCNB1 expression may be partially attributed to anti-tumor immune activity. Notably, this gene showed a significant correlation with TIDE score and several immune checkpoint molecules (HMGB1, CD80, IL13, ENTPD1, BTN3A1, TGFB1, VEGFA, ICOSLG, ICAM1, BTN3A2, TNFRSF14, CX3CL1, TLR4, ADORA2A, TNFRSF18) (Fig. 4D, E), indicating that CCNB1 might be an immunological regulatory target.

### Expression analysis in multiple datasets and clinical samples

**Fig. 5:**
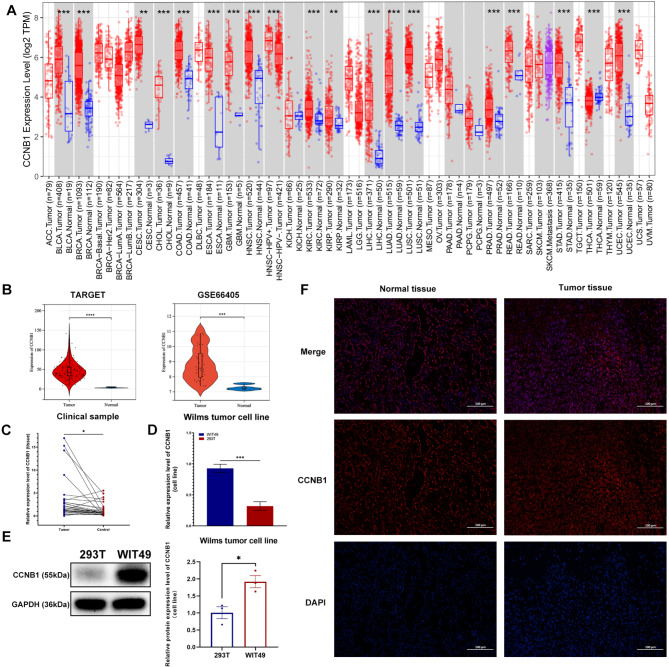
**CCNB1 exhibits a tumor-specific expression pattern. (A)** Pan-cancer analysis showed CCNB1 was highly expressed in a variety of tumors. **(B)** Two independent databases verified that CCNB1 was significantly overexpressed in WT. **(C and D)** RT-PCR verified that CCNB1 was highly expressed in WT tissues and cell lines. **(E and F)** WB and immunofluorescence were used to demonstrate the expression of CCNB1 at the protein level. * *p* < 0.05, ** *p* < 0.01, *** *p* < 0.001, **** *p* < 0.0001

To verify the key role of this gene, we evaluated CCNB1 expression levels in two different datasets. The results showed that this gene showed tumour-specific expression levels in two independent datasets (Fig. 5B). Pan-cancer analysis showed consistent results (Fig. 5A), suggesting this gene may be a key oncogene. Next, we performed RT-PCR in 37 WT cancer tissues and paired adjacent normal kidney tissues to verify the expression of CCNB1. The results showed that CCNB1 was significantly overexpressed in WT tissues compared with paraneoplastic tissues (Fig. 5C). Similarly, the expression of CCNB1 was significantly higher in the WT cell line WIT-49 than in the renal epithelial normal cell line 293T (Fig. 5D). Moreover, WB and IF results also showed that CCNB1 was highly expressed in WT cell lines and cancer tissues, respectively (Fig. 5E, F).

### Silencing of CCNB1 significantly inhibited the Proliferation, Migration, and Invasion ability of WT cells

**Fig. 6:**
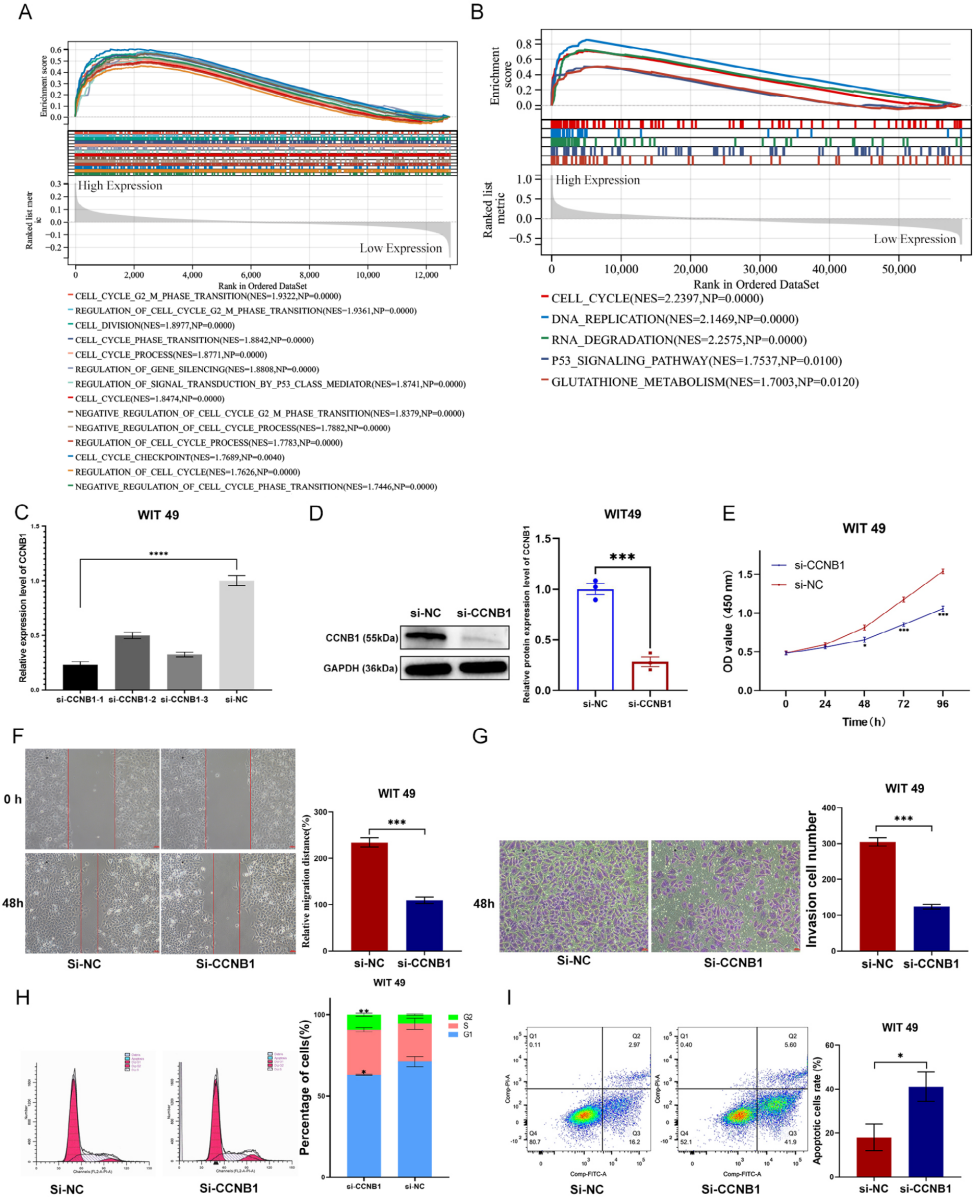
**CCNB1 promotes the malignant biological behavior of Wilms tumor cells via multiple phenotypes. (A-B)** single-gene GSEA enrichment analysis showed that CCNB1 was mainly involved in regulating cell cycle-related processes. **(C)** RT-PCR analysis of CCNB1 expression in WIT-49 cells after transfection with siRNAs. **(D)** Western blot analysis of CCNB1 expression in WIT-49 cells after transfection with si-CCNB1-1. **(E-G)** CCK-8 assay, cell Migration assay, and Transwell assay were used to demonstrate that silencing CCNB1 could significantly inhibit the proliferation, migration, and invasion ability of WIT-49 cells. **(H and I)** Flow Cytometry identified that silencing CCNB1 caused G2 phase arrest and promoted WIT-49 cell apoptosis. * *p* < 0.05, ** *p* < 0.01, *** *p* < 0.001, **** *p* < 0.0001

Single-gene GSEA enrichment analysis further suggested that this gene may be involved in biological processes such as regulation of ‘cell cycle’, ‘DNA replication’ (Fig. 6A, B). To demonstrate the role of CCNB1 in WT cells, three siRNA segments targeting CCNB1 were introduced into WIT-49 cells. After transfection for 48 h, we found that the knockdown efficiency of the first segment of si-CCNB1 was the highest (Fig. 6C). When compared to the si-NC group, si-CCNB1 protein expression was considerably lower in WIT-49 cells (Fig. 6D). Furthermore, cell function studies demonstrated that silencing this gene greatly reduced WIT-49 cell proliferation, migration, and invasive capacity (Fig. 6E-G).

In addition, this gene belongs to the family of cell cycle proteins. Therefore, we further verified whether this gene is involved in regulating the cell cycle and apoptosis levels of WIT-49 cells. Relatively perspective, silencing this gene caused G2 phase arrest (Fig. 6H) and promoted the apoptosis of WIT-49 cells (Fig. 6I) by flow cytometry.

## Discussion

Wilms tumor is the most prevalent malignant solid tumor in children’s urology, and the exact pathophysiology is unknown [33]. Despite advances in clinical therapy for WT, overall survival has not increased considerably, which can be related to a paucity of molecular markers for successful diagnosis and treatment. As a result, it is critical to investigate WT molecular markers in order to increase patient survival. Researchers may now integrate several bioinformatics methodologies to extensively investigate the main pathophysiology and clinical diagnosis or prognosis of many diseases at the molecular level, thanks to the fast growth of diverse bioinformatics databases and high-throughput studies [34]. Therefore, the integration of RNA sequencing data and mining in the database has become an important means to explore the pathogenesis of WT and speculate the possible markers for diagnosis and treatment.

In this study, we integrated and analyzed the high-throughput sequencing data of WT tissues and adjacent normal tissues, and found that most of the differential genes were up-regulated. GO and KEGG pathway analysis showed that DEGs were enriched in multiple pathways related to malignant biology, including ‘DNA replication’, ‘cell cycle’. To further identify key genes that play important roles, we established a PPI network based on the STRING database and screened 10 HUB genes. CCNB1 was found to be a risk factor for poor prognosis in WT patients by univariate COX analysis. Multifactorial analysis similarly demonstrated that this gene was an independent risk factor. This suggests a potential biological role for CCNB1 in WT progression. It has been shown that CCNB1 is a prognostic factor for overall survival and metastasis-free survival in breast cancer [35]. Several studies have confirmed that CCNB1 can be used as a diagnostic or prognostic biomarker for rhabdomyosarcoma, hepatocellular carcinoma, and meningioma [36–38]. Critically, our results of CCNB1 validation from transcriptome and protein levels and combined with clinicopathological information showed that CCNB1 was significantly highly expressed in WT. Functional experiments confirmed that silencing CCNB1 could inhibit the proliferation, invasion, and migration of WT cells, suggesting a crucial role of this gene in WT. Interestingly, there have been reports confirming the carcinogenic effect of high expression of CCNB1 in various cancers, including renal cancer, breast cancer, pancreatic cancer, hepatocellular carcinoma, and cervical cancer [39–42]. Several studies have shown that CCNB1 is a potential target for tumour intervention [43, 44]. CCNB1 has been reported to be considered other potentially useful genes for targeting hepatocellular carcinoma [45, 46]. Therefore, it is speculated that this gene may be a potential key therapeutic target for WT.

The cell cycle-related factor CCNB1 belongs to the family of cell cycle proteins [47]. It is well known that one of the distinguishing features of cancer is cell cycle dysregulation, leading to the unrestricted proliferation of cancer cells [48, 49]. In this study, GSEA enrichment analysis suggested that the gene promoted tumour progression by regulating the cell cycle, and further experiments proved that silencing the gene caused cell cycle G2 arrest and apoptosis. These findings encourage us to speculate that CCNB1 is essential for cell cycle progression and proliferation. To sum up, CCNB1 may regulate WT tumour progression through the cell cycle pathway.

Cancer patients usually have a large number of T cells, but most of them have lost their function [50]. One study found that CCNB1 caused T cell-dependent antibody responses in patients with cancer and precancerous lesions, suggesting that this gene is an important player in the immune control of tumour growth [51]. In addition, CCNB1, which is aberrantly expressed in patients with breast, lung, head, and neck cancers, can be recognized by antibodies and T cells as tumour antigens [52]. CCNB1 was discovered by Kao et al. as a common human epithelial tumor-associated antigen recognized by T lymphocytes [53]. Latner et al. [54] elucidated the enhanced expression of CCNB1 in virus-specific memory CD8^+^ T cells. It is worth noting that another important finding of our study is that CCNB1 is associated with immune scores and multiple immune cell infiltration levels. Single gene GSEA analysis suggests that the gene may be involved in the regulation of a variety of key immune cells, which may affect tumour immunity and lead to poor prognosis and can be used as an important indicator of cancer prognosis.

We demonstrated that CCNB1 is a promising prognostic marker and potential therapeutic target for WT by high-throughput sequencing combined with bioinformatics analysis and experimental validation. Despite the large sample bioinformatics analysis and clinical sample validation performed in this study, certain limitations remain: First, our prognostic analysis and model are based on a public cohort, and retrospective innate characteristics hinder clinical applicability. Therefore, prospective studies are needed for further validation before clinical application. Second, the downstream mechanism by which CCNB1 exerts its oncogene function remains unclear requiring further in vivo and in vitro experiments for validation. Third, the immune regulatory function of this gene in the tumour microenvironment is based on bioinformatics analysis, and the exact mechanism needs to be further explored.

In conclusion, our findings imply that CCNB1 is a significant prognostic biomarker and a possible therapeutic target for Wilms tumor.

### Electronic supplementary material

Below is the link to the electronic supplementary material.

Supplementary Material 1: KEGG Copyright Permission

Supplementary Material 2: Western blots of CCNB1 and GAPDH

## Data Availability

The datasets generated during and/or analyses during the current study are available in the TARGET and GEO Database. The datasets presented in this study can be found in online repositories. The name of the repository and accession numbers can be found below: NCBI; GSE31403 and GSE66405. We intend to share individual deidentified participant data. RNA sequencing data from eight tumor samples and paired normal tissue samples were uploaded to the gene expression omnibus database (https://www.ncbi.nlm.nih.gov/geo/, under the accession: GSE197047). you can access it now and it’s valid forever.

## Abbreviations

WT: Wilms tumour
COG: Children Oncology Group
TIDE: Tumour Immune Dysfunction and Exclusion
