# Supplementary material for: CCNB1 is a novel prognostic biomarker and promotes proliferation, migration and invasion in Wilms tumor
Source: BMC Med Genomics. 2023 Aug 17;16:189. doi: 10.1186/s12920-023-01627-3 (PMC10433552; doi:10.1186/s12920-023-01627-3)
Supplement: Supplementary file 1 — Supplementary Material 1: KEGG Copyright Permission [file 12920_2023_1627_MOESM1_ESM.pdf]

KEGG Database Project
Kanehisa Laboratories
www.kanehisa.jp

Ref: 230889

Permission is granted to BMC Medical Genomics to publish under the CC BY 4.0 open access license the following KEGG orthology image in the article "CCNB1 is a novel prognostic biomarker and promotes proliferation, migration and invasion in Wilms Tumor" written by Feng Liu and colleagues:

- K05868 G2/mitotic-specific cyclin-B1
  (RefSeq) CCNB1, CCNB (hsa:891)

subject to the condition that the original source is acknowledged by citing at least one KEGG paper.

Permission granted: Naomi Takahashi

Naomi Takahashi, Kanehisa Laboratories

Date: 26 May 2023

Copyright holder: Kanehisa Laboratories
