# Supplementary figures and images for: CCNB1 is a novel prognostic biomarker and promotes proliferation, migration and invasion in Wilms tumor
Source: BMC Med Genomics. 2023 Aug 17;16:189. doi: 10.1186/s12920-023-01627-3 (PMC10433552; doi:10.1186/s12920-023-01627-3)

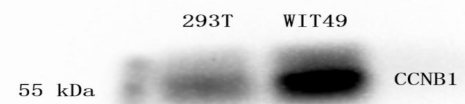

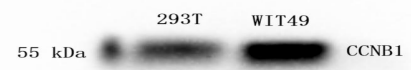

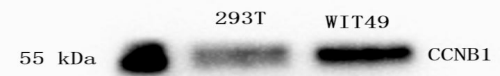

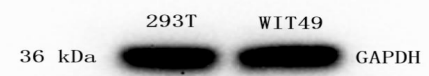

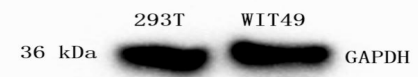

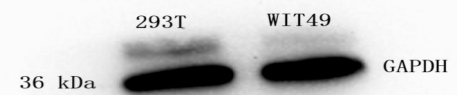

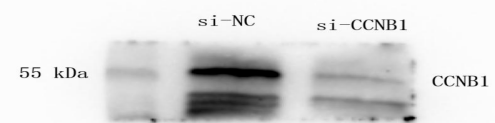

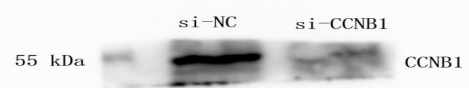

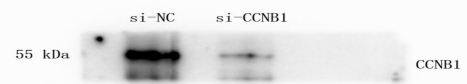

36 kDa si-NC si-CCNB1 GAPDH

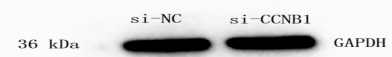

| Lane     | Protein | Approx. Molecular Weight (kDa) |
|----------|---------|--------------------------------|
| si-NC    | GAPDH   | 36                             |
| si-CCNB1 | GAPDH   | 36                             |

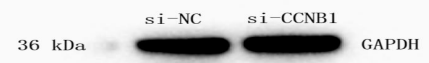

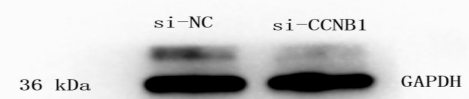

Supplement: Supplementary file 2 — Supplementary Material 2: Western blots of CCNB1 and GAPDH [file 12920_2023_1627_MOESM2_ESM.pdf]
